# Intraperitoneal bleeding after balloon-occluded retrograde transvenous obliteration: a case report

**DOI:** 10.1186/s13256-015-0546-3

**Published:** 2015-03-20

**Authors:** Yasuaki Furue, Hisashi Hidaka, Kaoru Fujii, Keiji Matsunaga, Wasaburo Koizumi

**Affiliations:** 1 Department of Gastroenterology, Internal Medicine, Kitasato University School of Medicine, Kitasato University Hospital, 1-15-1 Kitasato, Minami-ku, Sagamihara, Kanagawa 252-0374 Japan; 2 Department of Diagnostic Radiology, Kitasato University School of Medicine, Sagamihara, Japan

**Keywords:** Hepatic encephalopathy, Hepatic vein, Portal vein, Shunt

## Abstract

**Introduction:**

Hepatic encephalopathy is an important underlying cause of consciousness disorders. Possible causes of hepatic encephalopathy include hepatic failure and shunt encephalopathy resulting from a portosystemic venous shunt. Balloon-occluded retrograde transvenous obliteration is generally an effective treatment for hepatic encephalopathy.

**Case presentation:**

A 73-year-old Japanese woman was referred to our department because of disturbance of consciousness. Hepatic venous angiography disclosed a shunt between her left hepatic vein and her portal vein. The shunt was closed with the use of coils and N-butyl 2-cyanoacrylate. One hour after the procedure, she lost consciousness. The bleeding was ascribed to catheter-induced vascular injury. Emergency angiography was performed, and hemostasis was achieved with coils.

**Conclusion:**

Although bleeding is relatively rare after balloon-occluded retrograde transvenous obliteration, postoperative intraperitoneal bleeding is a serious complication.

## Introduction

Hepatic encephalopathy is an important underlying cause of consciousness disorders and should be included in the differential diagnosis [1]. Possible causes of hepatic encephalopathy include hepatic failure and shunt encephalopathy resulting from a portosystemic venous shunt [1,2]. Balloon-occluded retrograde transvenous obliteration (BRTO) is generally an effective and safe treatment for hepatic encephalopathy [3-6]. Here we report a case of intraperitoneal bleeding that occurred after BRTO.

## Case presentation

A 73-year-old Japanese woman presented disturbance of consciousness and wooziness. Her history included an atrial septal defect (ASD), tricuspid valve insufficiency, and atrial fibrillation. Closure of the ASD and tricuspid annuloplasty had been performed in October 1998. In February 2011, abdominal ultrasonography revealed a portosystemic venous shunt. She had been experiencing progressive bouts of wooziness since September 2012 and consulted a local physician. Hepatic encephalopathy was suspected, and she was referred to our hospital. Her blood ammonia level was 187μg/dL (normal range: 12 to 66μg/dL), suggesting that the wooziness was caused by hyperammonemia. Contrast-enhanced computed tomography (CT) showed a shunt involving the lateral superior branch of the portal vein (P2), the lateral inferior branch of the portal vein (P3), and the hepatic vein. She was admitted to undergo shunt closure by BRTO.

She had no history of cigarette smoking or drinking. Her Japan Coma Scale was 1, her blood pressure was 132/81mmHg, and her pulse rate was 93 beats/minute. A physical examination revealed no distinct abnormalities. Her platelet count was decreased, but there were no liver-function abnormalities potentially related to the hyperammonemia (Table 1). The results of tests for hepatitis B surface antigen and hepatitis C virus antibodies were negative. Viral hepatitis was, therefore, ruled out.

**Table 1:** **Laboratory data before the balloon-occluded retrograde transvenous obliteration procedure**

WBC	3000	/μL	CK	86	IU/L
RBC	32,900	/μL	T.Cho	134	mg/dL
Hb	11.7	g/dL	TG	28	mg/dL
Ht	35.8	%	Glu	75	mg/dL
Plt	97,000	/μL	BUN	12.9	mg/dL
PT-%	55.2	%	Cr	0.56	mg/dL
aPTT	32.4	Seconds	NH3	109	μg/dL
TP	6.5	g/dL	Na	141	mEq/L
Alb	3.4	g/dL	K	4.2	mEq/L
T.Bil	1.3	mg/dL	Cl	108	mEq/L
D.Bil	0.6	mg/dL	Ca	9.1	mg/dL
AST	24	IU/L	CRP	<0.1	mg/dL
ALT	20	IU/L	AFP	3.7	ng/mL
LDH	264	IU/L	DCP	18	mAU/mL
ALP	178	IU/L	HBsAg	(−)	
γ-GTP	36	IU/L	HCV-Ab	(−)	
AMY	70	IU/L			

AFP: alpha-fetoprotein; Alb: albumin; ALP: alkaline phosphatase; ALT: alanine aminotransferase; AMY: amylase; aPTT: activated partial thromboplastin time; AST: aspartate aminotransferase; BUN: blood urea nitrogen; Ca: calcium; CK: creatine kinase; Cl: chloride; Cr: creatinine; CRP: C-reactive protein; D.Bil: direct bilirubin; DCP: des-gamma-carboxy prothrombin; γ-GTP: γ-glutamyl transpeptidase; Glu: glucose; Hb: hemoglobin; HBsAg: hepatitis B surface antigen; HCV-Ab: hepatitis C virus antibodies; Ht: hematocrit; K: potassium; LDH: lactate dehydrogenase; Na: sodium; NH3: ammonia; Plt: platelet; PT-%: prothrombin time percent; RBC: red blood cell; T.Bil: total bilirubin; T.Cho: total cholesterol; TG: triglycerides; TP: total protein; WBC: white blood cell.

**Figure 1:**
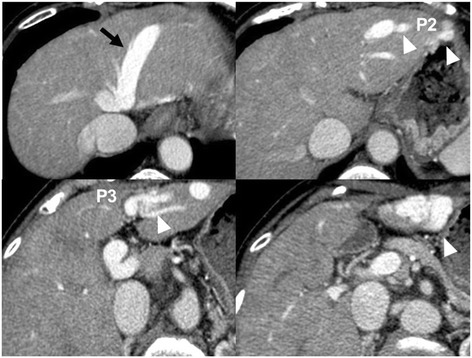
**Abdominal contrast-enhanced computed tomography.** Abdominal contrast-enhanced computed tomography shows shunts of the lateral superior branch (P2) and the lateral inferior branch of the portal vein (P3) with the left hepatic vein (black arrow). The left hepatic vein had anastomosed to P2 and P3 via shunts (white arrowheads).

**Figure 2:**
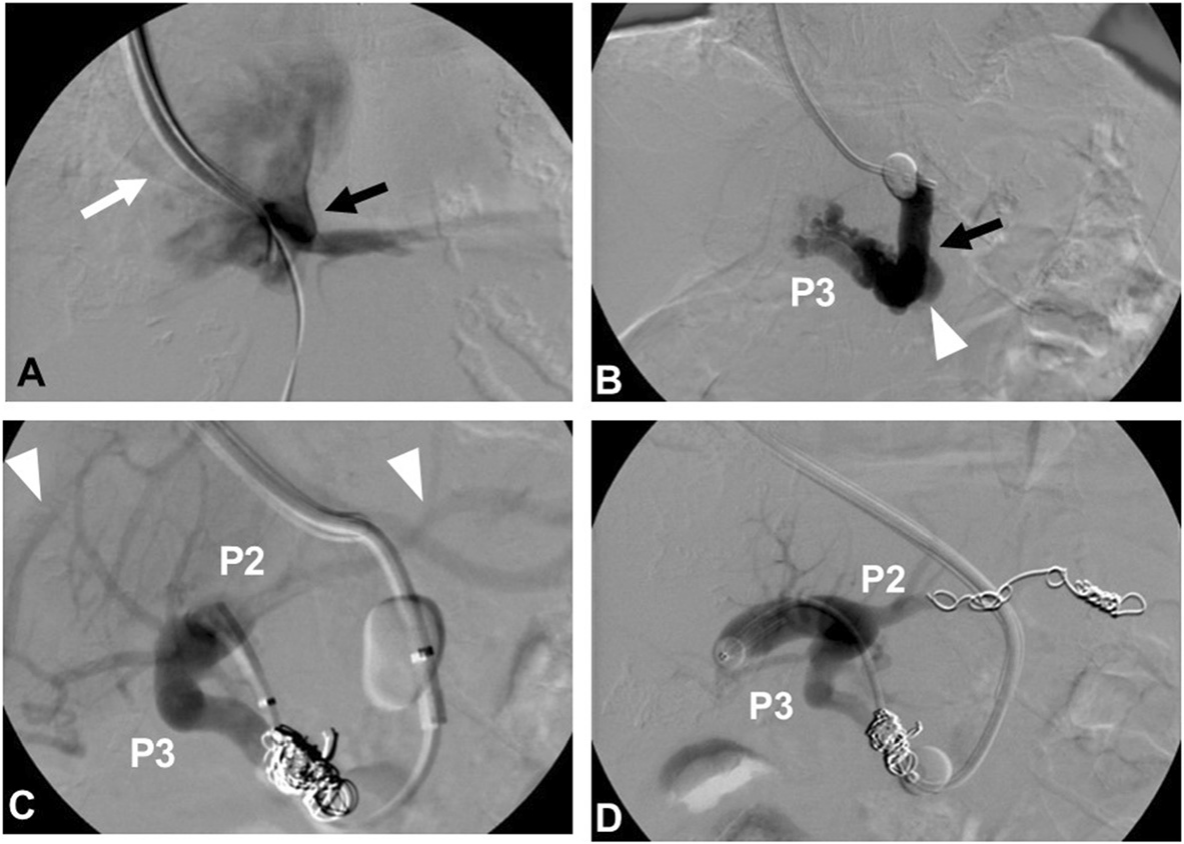
**Balloon-occluded retrograde transvenous obliteration of the intrahepatic portosystemic shunt was performed using N-butyl-2-cyanoacrylate and embolization coils. (A)** The left inferior phrenic vein (white arrow) merged into the left hepatic vein (black arrow). **(B)** The left hepatic vein (black arrow) via a shunt (size: 10mm; white arrowhead) merged with the lateral inferior branch of the portal vein (P3). The left hepatic vein was occulted transiently with a balloon. **(C)** After the shunt was embolized with coils, the lateral superior branch of the portal vein (P2) also merged with the left hepatic vein via other shunts (size: 6mm; white arrowheads). **(D)** The shunt was embolized using coils.

Contrast-enhanced CT showed a shunt involving her left hepatic vein, P2, and P3 (Figure 1). Upper gastrointestinal endoscopy showed no evidence of esophagogastric varices. After admission, she was restricted to high-protein meals and received an amino-acid preparation. Her wooziness improved 2 days later, and her blood ammonia returned to a normal level (62μg/dL) within 1 week. Hyperammonemia due to a portosystemic venous shunt was diagnosed. Because she was elderly and had decreased cardiac function after closure of the ASD and tricuspid annuloplasty, surgical treatment was considered too dangerous. After explaining the situation to her and her family and receiving informed consent, BRTO was performed. An approach was made from her right internal jugular vein. Her left hepatic vein had anastomosed to the left inferior phrenic vein (Figure 2A). The contrast medium showed that her left hepatic vein had anastomosed to P3 via a shunt (size: 10mm), thus forming a loop-like route (Figure 2B). Embolization was performed using coils on the portal vein side of this route (Figure 2C). Subsequent angiography of P2 confirmed the presence of a shunt (size: 6mm) and the blood flow to her hepatic vein. Embolization was performed using coils on the portal vein side of the shunt (Figure 2D). Coils were again emplaced to P3, and fine branches were embolized using a mixture of N-butyl-2-cyanoacrylate (NBCA) and iodized oil (ratio, 1:3). In addition, coils were emplaced in the portal vein side of the shunt involving P3 and her left hepatic vein. Angiography via the route last approached revealed the blood flow from P3 to her left hepatic vein. Therefore, coils were placed at the hepatic vein side of the shunt involving P3 and her left hepatic vein. Embolization was performed with the use of NBCA. After embolization, the lack of movement of contrast medium in her hepatic vein was confirmed completing the procedure.

**Figure 3:**
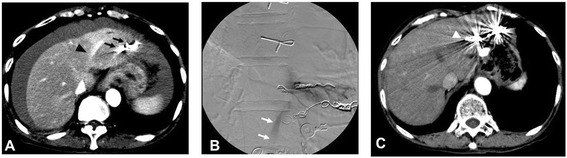
**Contrast-enhanced computed tomography and angiography. (A)** Contrast-enhanced computed tomography to diagnose bleeding. Remarkable leakage of contrast medium from the left inferior phrenic vein was seen (black arrowhead). There was excessive blood around the liver. There were coil embolizations of shunts (black arrows). **(B)** Angiography revealed leakage of contrast medium (white arrows) from the left inferior phrenic vein. **(C)** Contrast-enhanced computed tomography 2 months later. There were no ascites around the liver. There were coils for hemostasis (white arrowhead).

**Figure 4:**
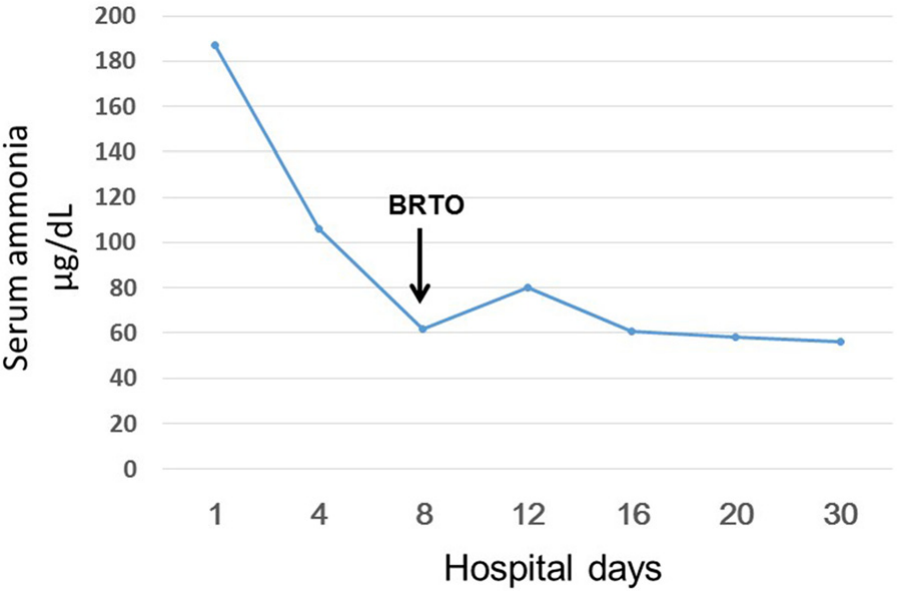
**Course of the blood ammonia level.** BRTO: balloon-occluded retrograde transvenous obliteration.

After returning to the ward, she presented with paleness, hypotension (systolic blood pressure, 48mmHg), and an increased pulse rate of 120 beats/minute. Intraperitoneal bleeding was suspected, so contrast-enhanced CT was performed. Slight leakage of contrast medium from her left inferior phrenic vein was seen (Figure 3A). Angiography revealed leakage of contrast medium from her left inferior phrenic vein (Figure 3B). Embolization was, therefore, performed using NBCA and embolization coils. The procedure was performed while she received a blood transfusion. On the day after the vascular embolization, her hemoglobin level returned to her normal level. Because her state of shock did not improve, she was intubated and admitted to our intensive care unit. She responded to the blood transfusion, supplementation of coagulation factors, and artificial ventilation management; the acute phase resolved. The intraperitoneal hematoma also improved (Figure 3C). Subsequently, pleural effusion developed in association with elevated portal vein pressure and chronic heart failure but improved after treatment with an oral diuretic (furosemide 20mg/day) and beta-blocker (carvedilol: 10mg/day). She had general muscle atrophy, particularly of her skeletal muscles due to the extensive hospitalization. Therefore, she had physical therapy rehabilitation for 1 month. She received treatment in our hospital for 3 months and was then discharged. She subsequently visited our hospital every 2 months after the treatment for 2 years. She has never since presented with any symptoms of hepatic encephalopathy (Figure 4).

## Discussion

To the best of our knowledge, this is the first report in which intraperitoneal bleeding occurred after BRTO, which is generally an effective treatment for hepatic encephalopathy and gastric varices [3-6]. In a Japanese national survey the incidence of complications after BRTO (in patients with a prolonged hospital stay) was only 1.4% [3]. The most common complication was aggravation of liver dysfunction in 0.66% of patients, followed by pulmonary failure in 0.17%, gastrointestinal bleeding in 0.1%, heart failure in 0.06%, and aggravation of renal dysfunction in 0.06% [3]. Therefore, BRTO is considered to be a safe procedure.

In the present case, intraperitoneal bleeding might have been caused by vascular injury due to manipulation of the catheter, which might have strayed into the left inferior phrenic vein during BRTO. The left inferior phrenic vein was not used for shunt embolization in the present patient. Blood flow from the left inferior phrenic vein might have been blocked by the inflated balloon, thereby precluding detection of the vein intraoperatively. After returning to the ward, the patient had hypotension and an increased heart rate, and intraperitoneal bleeding was diagnosed with CT. Transcatheter embolization was performed under angiographic guidance for hemostasis. Although the present case was extremely rare, such complications present a very serious threat. Imaging examinations such as CT should, therefore, be performed after BRTO. To provide safer therapy, indications for treatment (evaluation of hepatic function), and treatment response should be carefully evaluated. On the basis of our experience with the present patient, our hospital now requires that CT be performed after BRTO on the day of the procedure.

## Conclusions

BRTO is currently recognized as a relatively safe and minimally invasive treatment. Intraperitoneal bleeding after BRTO is relatively rare but serious; therefore, caution is required, for example our hospital now requires a CT examination after BRTO on the day of the procedure.

## Consent

Written informed consent was obtained from the patient for publication of this case report and any accompanying images. A copy of the written consent is available for review by the Editor-in-Chief of this journal.

## Abbreviations

ASD: Atrial septal defect
BRTO: Balloon-occluded retrograde transvenous obliteration
CT: Computed tomography
NBCA: N-butyl-2-cyanoacrylate
P2: Lateral superior branch of the portal vein
P3: Lateral inferior branch of the portal vein
